# Removal of Left Superior and Check for Sarcoma

**Published:** 1883-01

**Authors:** George Lawson

**Affiliations:** SURGEON TO MIDDLESEX HOSPITAL, ETC.


### ARTICLE VII.

### *Sarcoma of Left Superior Maxilla and Cheek—Removal and Recurrence.*

BY GEORGE LAWSON, F. R. C. S., SURGEON TO MIDDLESEX HOSPITAL, ETC.

On June 12th, 1882, E. A.—æt. 18, a domestic servant, was admitted into Regent Ward for growth of upper jaw, with following history:

Christmas last she suffered with severe pain in region of molar teeth of upper jaw (left), extending over side of face; was told by medical attendant that it was due to cutting of wisdom teeth.

In the following January a swelling was first noticed in left cheek which rapidly increased, she therefore was admitted into St. Bartholomew's Hospital, Rochester, when an incision was made into left cheek and tumor was removed. The growth at that time was larger than at present.

Erysipelas followed the operation, but she made a good recovery.

Six weeks ago she first noticed swelling again in left cheek at site of operation, which increasing she applied for admission, and presented following conditions:

A fairly well-nourished young woman, complaining of pain and swelling of left cheek, which is much enlarged, and presents a cicatrix the result of previous operation. A hard lump is felt superficially about the size of a walnut, which is connected with a similar mass attached to left superior maxilla. The mouth can only be opened to a small extent; teeth are perfect in both jaws. The roof of hard palate on left side is seen to bulge into mouth, and there is some swelling about the alveolar process; left nostril is completely blocked up. The left eye is pushed upwards and outwards, the sight of which is destroyed. There were no enlarged glands.

On June 14th, Mr. Lawson completely excised the left supra maxilla in the usual way, and also removed that portion of the growth which had invaded the cheek; actual cautery was then applied to the deep parts of the wound and to the cheek. Chloroform was administered but not to complete anæsthesia.

On examining growth it was found to be rather soft in consistence, of a pale, yellowish-white colour and encapsuled, and presented a lobulated cauliflower appearance.

Microscopically it was seen to be composed chiefly of small round cells, but in places there was a considerable growth of spindle-cells and fibrous tissue.

Patient rapidly recovered, on July 1st wound was healing well, on the 6th was quite healed externally.

On July 11th she complained of great pain in front of left external auditory meatus, where a swelling was found. The sight of left eye was completely destroyed.

On July 25th she was discharged, there appeared to be a recurrence of the growth in the cheek and also in the hollow left by the removal of the supra maxilla.—*British Journal of Dental Science.*

